# Increasing temperature reduces the coupling between available nitrogen and phosphorus in soils of Chinese grasslands

**DOI:** 10.1038/srep43524

**Published:** 2017-03-07

**Authors:** Yan Geng, Frank Baumann, Chao Song, Mi Zhang, Yue Shi, Peter Kühn, Thomas Scholten, Jin-Sheng He

**Affiliations:** 1 Department of Ecology, College of Urban and Environmental Sciences, and Key Laboratory for Earth Surface Processes of the Ministry of Education, Peking University, 5 Yiheyuan Rd., Beijing 100871, China; 2 Department of Geoscience, Soil Science and Geomorphology, University of Tuebingen, Ruemelinstrasse 19–23, 72070 Tuebingen, Germany; 3 Odum School of Ecology, University of Georgia, 140 E Green St, Athens, GA 30602, USA; 4 Key Laboratory of Adaptation and Evolution of Plateau Biota, Northwest Institute of Plateau Biology, Chinese Academy of Sciences, 23 Xinning Rd., Xining 810008, China

## Abstract

Changes in climatic conditions along geographical gradients greatly affect soil nutrient cycling processes. Yet how climate regimes such as changes in temperature influence soil nitrogen (N) and phosphorus (P) concentrations and their stoichiometry is not well understood. This study investigated the spatial pattern and variability of soil N and P availability as well as their coupling relationships at two soil layers (0–10 and 10–20 cm) along a 4000-km climate transect in two grassland biomes of China, the Inner Mongolian temperate grasslands and the Tibetan alpine grasslands. Our results found that in both grasslands, from cold to warm sites the amounts of soil total N, total P and available P all decreased. By contrast, the amount of available N was positively related to mean annual temperature in the Tibetan grasslands. Meanwhile, with increasing temperature ratio of available N to P significantly increased but the linear relationship between them was considerably reduced. Thus, increasing temperature may not only induce a stoichiometric shift but also loose the coupling between available N and P. This N-P decoupling under warmer conditions was more evident in the Tibetan alpine grasslands where P limitation might become more widespread relative to N as temperatures continue to rise.

Nitrogen (N) and phosphorus (P) are the soil nutrients that most commonly limit terrestrial production^1^,^2^. Changes in environmental conditions could greatly influence soil nutrient cycling processes, potentially affecting soil N and P concentrations and their stoichiometry. Because of the different degrees of control exerted on the supply of N and P by biological and geochemical processes, the levels of soil N and P availability have been found to be differently affected by environmental changes^3^,^4^,^5^,^6^. The altered circulations of N and P would significantly affect the structure, functioning and diversity of terrestrial ecosystems^7^,^8^,^9^. Understanding the changes in soil N and P status and dynamics along environmental gradients, particularly along climatic gradient that plays the most important role in shaping the physical landscape, is important for understanding the underlying patterns of nutrient fluxes and the biogeochemical mechanisms of the response of terrestrial ecosystem to climate changes.

Temperature is the most important climatic factors controlling soil N and P cycles. An increase in temperature generally facilitates the decomposition of soil organic matter and accelerate the accumulation of soil available nutrients^10^. Other research, however, indicated that warming had no noticeable influence on the rate of net mineralization of N and P^11^,^12^,^13^. In some regions, increased temperature even reduces the nutrients availability in soil. For example, a warmer condition resulted in a decrease in the supply of inorganic N in US northern forests, because the positive effect of higher temperature on microbial activity might be overcome by warmer winter leading to a reduced snowpack, more soil frost and more soil freeze-thaw events^14^. More importantly, existing evidence showed that the availability of N and P in soils may not necessarily vary in unison with changing temperature. For instance, across temperate steppes in northern China, while soil available N decreased from cool to warm sites, soil P availability increased with temperature^15^. Other experimental warming treatments have also been shown to affect the relative availability of soil N with respect to P^13^,^16^. As a result, increasing temperature would cause changes in the absolute amount of N and P in soil as well as in their relative proportions. The different direction and magnitude of soil N and P in response to temperature change might result in a N-P imbalance, which could in turn influence vegetation growth, community composition and ecosystem function^17^,^18^,^19^. However, the influence of temperature regime on elemental balance has received little attention.

Grasslands cover about one-third of Earth’s land surface and naturally encompass a broad climatic gradient. In China, grasslands cover more than 40% of the total land area, providing an exceptional opportunity to study the spatial patterns and drivers of soil nutrient availability along large-scale gradients of climate. In particular, the Tibetan plateau is the highest and largest alpine grassland region in the world. The distinct geothermal gradient and hydrological cycle on the Plateau could exert significant effects on the biogeochemical processes regulating soil N and P cycles, which might differ from those in other regions^20^. Meanwhile, variations of soil N and P may display different trends with the alpine climate. For example, along an alpine grassland transect across Northern Tibet, as temperature increased from southern to northern regions, soil total N stocks declined. In contrast, distribution of soil total P did not reflect the role of temperature in controlling P supply from mineral sources^21^. Over the last several decades, the Tibetan alpine grassland has experienced a significant temperatures rising of 0.16 °C per year^22^. This warming situation might upset soil N-P balance and result in a stoichiometric shift in soil N:P ratio^23^. While recent studies have assembled considerable data on the storage of soil N and P across Chinese grassland biomes^24^,^25^,^26^,^27^, the extent of temperature change effects on soil N and P availability remains unclear. Moreover, few studies have considered this topic from a stoichiometric perspective, i.e., examining the coupling relationships between N and P (N:P ratios).

Here, we conducted a 4,000 km transect analysis with soil measurements to evaluate how increasing temperature affect soil N-P balance in China’s grassland biomes, including two grassland biomes, the temperature grasslands on the Inner Mongolian Plateau and the alpine grasslands distributed on the Tibetan Plateau. The unique features of this transect include a continuum of mesic to xeric grassland types, diverse soil types, distinct patterns of temperature and precipitation, and relatively light human disturbance. Temperature controls on the biogeochemical cycles of soil elements are particularly relevant for this transect because in the alpine grassland biological activity is mainly constrained by the very low growing season temperature. We examined if there were predictable changes in soil total and available N and P contents associated with changes in temperature; whether the changes in pools of N and P were same in magnitude and subsequently whether there was a stoichiometric shift in N:P ratio. As the difference in available N (and P) between the two grassland biomes can be attributed to the differences in nature of their parent materials, development stages and patterns of their parent total N (and P), we also calculated the proportion of soil available relative to total N (and P), which may be more appropriate for characterizing soil nutrient availability than the absolute values of available N and P concentrations^28^. Because available N is derived mainly from organic matter decomposition but the level of available P is a joint outcome between weathering of parent material and biotic processes, we hypothesized that higher temperature may lead to higher N availability (and higher available to total N ratios) due to enhanced microbial activity, while soil P availability (and available to total P ratios) should be less affected by temperature. As a result, ratio of available N:P would be likely to increase. Moreover, we expected that the increased amount of available N from accelerated N mineralization would promote vegetation growth, which may stimulate plant uptake of P and result in a reduction in the amount of soil P. These processes tended to weaken the coupling between soil N and P. In addition, we also predicted that the alpine soils may be more responsive to changes in temperature as the cold stress on biological processes could be relieved under warmer conditions. Furthermore, since vegetation cover and soil properties both mostly reflect the long-term effect of climate, it is also of interest to study how these variables affect soil N and P, whether in similar or different ways.

## Results

### Effect of increasing temperature on soil total and available N and P

At the site level, the abundance of soil N and P varied greatly between the temperate and alpine grasslands. Mean total N concentration in Tibetan grasslands was approximately three times higher than in Inner Mongolia (Table 1). Soils of Tibetan Plateau also showed higher total and available P-values. However, available N contents were similar between these two regions (Table 1). These patterns were also evident for area-based measurements (Table S1). Compared with Inner Mongolian grasslands, the Tibetan alpine grasslands were higher in total N:P ratio but lower in available N:P ratio (Table 1).

**Figure 1:**
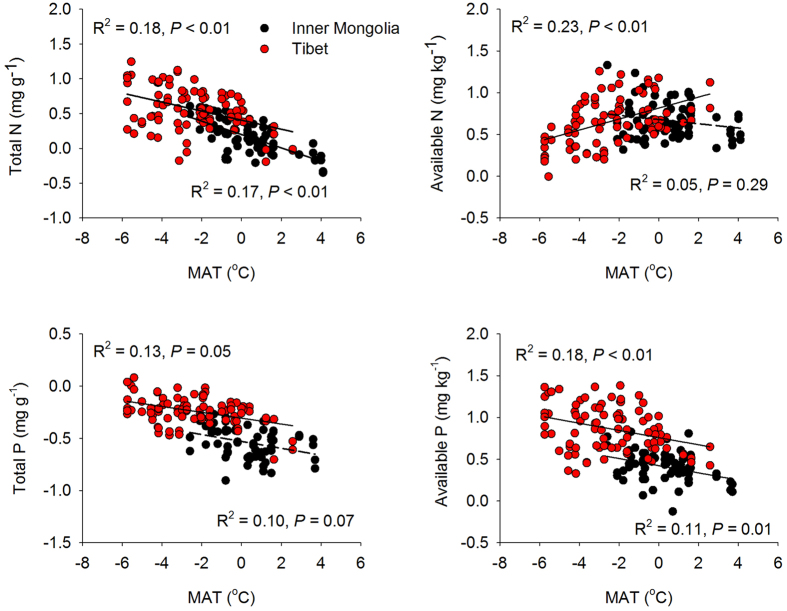
Soil N and P in relation to mean annual temperature. The solid lines represent significant linear regressions (*P* < 0.05) and the short dashed lines represent insignificant regressions (*P* > 0.05). The values of soil N and P are log-transformed. R^2^, proportion of variance explained. MAT, mean annual temperature.

Plotting soil nutrients against temperature revealed some significant relationships. With increasing temperature from cold to warm sites, soil total N, total P and available P all decreased, in both alpine and temperate grasslands (Fig. 1a,c,d). By contrast, soil available N was increased with MAT in Tibetan grassland and insignificantly related to temperature for sites in Inner Mongolia (Fig. 1b). Correlations between total and available N and MAT (Fig. 1a,b) were higher than the correlations between total and available P and MAT (Fig. 1c,d). Meanwhile, the correlations between available N and P and MAT (Fig. 1c,d) were higher than the correlations of total N and P with MAT (Fig. 1a,b). Plotting soil N and P against precipitation obtained nearly opposite trends (Fig. S1), however, most of the correlations were statistically insignificant, implying that temperature was a stronger driver for soil nutrients in our studied sites.

### Effect of increasing temperature on soil N and P stoichiometric ratios

**Figure 2:**
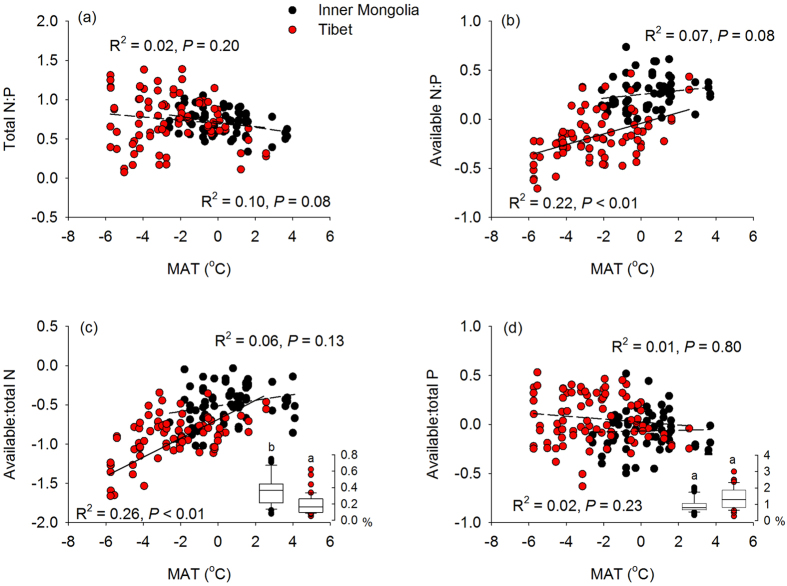
Ratios of soil total N:P, available N:P, available: total N and available: total P in relation to mean annual temperature. The solid lines represent significant linear regressions (*P* < 0.05) and the short dashed lines represent insignificant regressions (*P* > 0.05). The inside box plots showed the ratios of available: total N and available: total P in Inner Mongolian and Tibetan grasslands. The error bar indicates the standard error, and the bottom and top of the box are the first and third quartiles, and the band inside the box is the median. The N:P ratio values are log-transformed. MAT, mean annual temperature.

Neither the Tibetan nor Mongolian sites showed significant relationships between total N:P and MAT (Fig. 2a), whereas available N:P and MAT was positively correlated in the Tibetan grassland (*P* < 0.01) and marginally correlated in Inner Mongolian grassland (*P* = 0.08, Fig. 2b). Ratio of available to total N increased dramatically as MAT increased for alpine soils (Fig. 2c), however, this enhancement in soil N availability induced by higher temperature was not observed in Inner Mongolian grasslands (Fig. 2c). Ratio of available: total P was not a function of MAT, and the relationship even tended to be negative (Fig. 2d). Tibet had a lower ratio of available: total N compared with Inner Mongolia, whereas the ratio of available: total P did not differ significantly between these two regions (Fig. 2c,d). Precipitation did not have detectable influences on either total N:P or available N:P ratios (Fig. S2).

**Figure 3:**
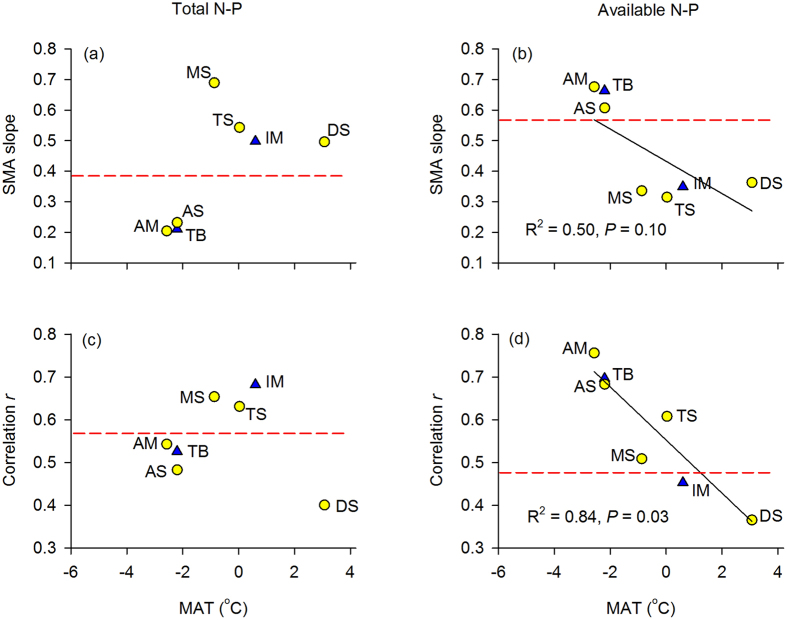
Standard major axis (SMA) slope and strength (*r*) of soil N-P relationships in relation to mean annual temperature at vegetation type and regional scales. Sites are displayed with different symbols: circles, vegetation type; triangles, region. Dashed horizontal lines indicate the SMA slopes or correlation *r*-values averaged for the whole-dataset relationships. IM, Inner Mongolia; TB, Tibet; AM, alpine meadow; AS, alpine steppe; DS, desert steppe; MS, meadow steppe; TS, typical steppe; MAT, mean annual temperature.

Overall, the Inner Mongolian temperate grasslands showed closer relationship between total N and P than the Tibetan alpine grassland, as reflected by the greater magnitude of the slope and higher correlation coefficient (*r*) (Fig. 3a,c). However, no clear trend existed between the correlation strength and MAT among the five vegetation types (Fig. 3a,c). On the contrary, for the linear relationship between available N and P, both SMA slope and *r* were higher in the Tibetan grassland than in Inner Mongolian grassland (Fig. 3b,d). At the vegetation type scale, the association between available N and P (*r*) was apparently reduced from alpine (alpine meadow and alpine steppe) to temperate (meadow steppe, typical steppe and desert steppe) grassland ecosystems (R^2^ = 0.84, *P* = 0.03, Fig. 3d), and the relationship between available N-P slope and MAT was marginally significant (R^2^ = 0.50, *P* = 0.10, Fig. 3b), implying a decoupling of soil available N and P with increasing MAT.

### Controlling factors for soil N and P

**Figure 4:**
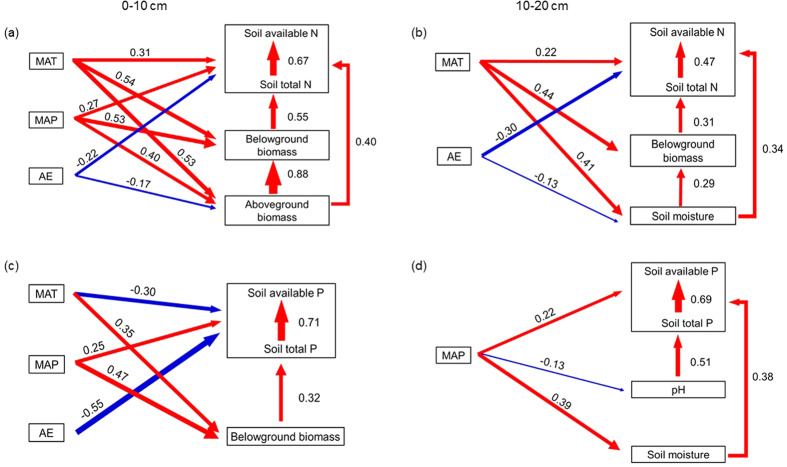
Structural equation models (SEM) of the ratios of soil available to total N and available to total P at soil depths of 0–10 cm and 10–20 cm. Red arrows represent positive correlations and blue ones represent negative correlations. Width of the arrows reflects the magnitude of the correlation between two variables. We report the path coefficients beside each arrow. Overall fit of SEM was evaluated using model Chi-square, P-values, comparative fit index (CFI) and root mean square error of approximation (RMSEA). (**a**): Chi-square = 0.728, P = 0.732, CFI = 1.000, RMSEA < 0.001; (**b**): Chi-square = 0.553, P = 0.758, CFI = 1.000, RMSEA < 0.001; (**c**): Chi-square = 1.794, P = 0.601, CFI = 1.000, RMSEA < 0.001; (**d**): Chi-square = 2.904, P = 0.470, CFI = 0.990, RMSEA = 0.003. These model fit parameters indicated that our models were of good fit. MAT, mean annual temperature; MAP, mean annual precipitation; AE, actual evapotranspiration.

Both climate change and climate-induced change in soils and vegetation affected soil N and P concentrations (Table 2). Climate data (temperature, precipitation and evapotranspiration) collectively explained 38–62% of the variation in soil N and P (Table 2). Plant above- and belowground biomass also contributed to a large fraction of soil N and P (10–44%). Soil physical properties (pH and moisture) were less important drivers over soil N and P, accounting for 10–38% of the influence (Table 2). MAT was positively related to ratio of available: total N for both depth increments (Fig. 4a,b) while only negatively related to ratio of available: total P at 0–10 cm (Fig. 4c). Temperature and precipitation also indirectly affected soil N and P through its effect on plant biomass (Fig. 4a,c), soil moisture (Fig. 4b,d) and pH (Fig. 4d). Ratio of available: total N increased with increasing plant above- and below-ground biomass (Fig. 4a). Soil P availability were less affected by plant biomass (Fig. 4c,d), especially at the deeper layer where soil moisture and pH became the main controlling factors of the ratio of available: total P.

## Discussion

Temperature is an important determinant of N mineralization and N availability in soils^29^,^30^. Cold climate has been shown to inhibit microbial activity, leading to slower N mineralization rates and hence lower N supply to plants^31^. A positive correlation between soil available N and MAT on the Tibetan Plateau could largely be a result of the increased soil microbial activity and microbial biomass under warmer conditions. In incubation experiments, N mineralization in the alpine soils has been found to accelerate with the rise of temperature^32^,^33^,^34^. On the contrary, soil N availability in Inner Mongolian grasslands was not significantly related to MAT, consistent with the results of a recent study^15^. Temperature may thus not be a limiting factor for N mineralization in the arid/semi-arid/dry sub-humid steppes, instead, the increasing temperature may exacerbate the drought effects by reducing soil water content and vegetation cover, both of which are likely to cause soil erosion and nutrient loss^35^. In contrast to available N, the amounts of total N did not increase with increasing temperature, probably because a warmer condition was associated with vigorous seasonal vegetation growth that increased the above- and belowground biomass, and hence the sequestration of nutrients was also enhanced. Therefore, a large accumulations of total N in soil may not occur, although a large amount of available N may remain after mineralization^13^.

In this study, increasing temperature reduced the amounts of both total and available P. First, unlike soil N, P supply in soil is governed by a combination of biological and chemical (adsorption/desorption and dissolution) processes. N and P cycling may thus respond differently to environmental factors such as temperature. For example, in subarctic soils of Northern Sweden, both elevation-associated changes in temperature^36^ and *in situ* soil incubation^37^ showed contrasting effects on the availability of soil N and P. Warming treatment resulted in proportionally large mineralization of N, in contrast to non-significant P mineralization^37^. Second, during growth, plant biomass N:P ratios are constrained to a certain range^38^. Some data showed that increased N inputs led to increases in both foliar N and P^39^, and the increasing uptake of N encouraged the absorption and utilization of soil P, resulting in a loss of soil P^11^,^40^,^41^. Moreover, enriched soil N availability generally reduces plant N resorption while P resorption could be less affected (or even promoted)^39^,^42^, leading to more N relative to P being returned to the soil through plant litter production. Meanwhile, plant litter P was difficult to breakdown because it existed in the form of phospholipid and phytate which must be processes by specialized enzymes and are affected by multiple factors^43^. As a result, P absorbed by plants could not soon be released into soil and the P availability may temporarily decreased. Furthermore, in soils with ample N supply, the relative rate of microbial immobilization of P was higher than the rate of mineralization, so that the amount of available P declined^44^.

The different responses of available N and P to temperature changes altered their coupling relationship. By compiling a large dataset from both historical national soil survey and regional soil survey across China’s grassland, Yang *et al*.^23^ found a widespread increase in topsoil total N:P ratios from the 1980 s to the 2000 s, suggesting that total N and P tend to be decoupled under changing environments. Other studies also reported increase in N:P ratios in soil during long-term ecosystem development^19^. This N-P decoupling may lead to ecosystem nutrient limitation from N towards P^1^,^45^. Traditionally, these research only measured soil total nutrients as an index of soil fertility while in fact soil available N and P may play a more important role for plant uptake. The decoupling of available N and P found in this study may thus have a more profound effect on the vegetation, as leaf N and P of grassland species are more strongly correlated with soil available N and P than with soil total N and P^46^. It is also noticeable that the correlation between available N and P was reduced from cold (alpine meadow and alpine steppe) to warm (meadow steppe, typical steppe and desert steppe) sites, strengthening the idea that available N and P might become less tightly coupled with increasing temperature. Given that N and P of alpine soils are highly correlated but respond inversely to temperature changes, it is reasonable to speculate that the Tibetan alpine grassland may be more vulnerable to the current trend of global warming. By contrast, increasing temperature may not have such a substantial effect on the circulation of N and P in temperate grassland ecosystems. Similar with our results, simulated warming experiment in temperate meadow steppe produced no apparent effect on the amounts of soil N or P (either in total or available forms), and soil total and available N:P ratios also remained relatively unchanged^13^.

Variations in soil N and P across the studied transect could also be attributed to the climate-induced change in vegetation and soil properties, as shown by the SEM model. The closer association of plant biomass with soil N availability, rather than with P availability, again implied that soil P supply is determined by the parent material, texture and other soil physiochemical processes in addition to the amounts of organic inputs. Moreover, the different responses of N and P availability to the environmental variables such as soil moisture and pH further demonstrated that N and P-related processes might become asynchronous with changing environment. However, it should be noted that N and P cycling are decoupled to some extent, but not separated, despite the stoichiometric shift in their ratios, as long as the biogeochemical cycles of N and P are coupled with primary production, respiration and decomposition in terrestrial ecosystems^47^.

Another striking fact is that available N pools in Tibetan and Inner Mongolian Plateau were similar, despite a wide difference in total N between the two regions. Interestingly, previous studies have found that plants growing on temperate and alpine grasslands had similar N concentrations in both leaves and fine roots^48^,^49^. Diverse evidence suggests that plant [N] and [P] might be dependent on soil fertility^2^,^50^,^51^,^52^. Then the consistent soil available N contents might partly result in the similar N concentrations in plant tissues between the two grassland biomes. We acknowledge that the acquisition of organic N is also widespread among plants from many ecosystems. These organic fractions could also be made available to plants by mycorrhizal fungi, or by nonmycorrhizal roots that take up amino acids with high affinity^53^. However, plants generally compete for a minor fraction of the total amino acid flux, and in most cases this do not form a significant N resource^54^.

## Conclusions

Through analyzing the topsoil N and P data of 80 sites obtained from a regional survey across grasslands in the Tibetan and Inner Mongolia Plateau, we highlighted that as MAT increases soil N availability was enhanced while P availability was reduced, resulting in a decline of available N:P ratio. Increasing temperature may not only cause a shift in soil available N:P stoichiometry but also lower the strength of available N-P correlation. This N-P decoupling under warmer conditions was more evident in the alpine grassland ecosystems. As the global temperatures are expected to continue to rise, N may become progressively more available to plants relative to P, resulting in a more widespread P limitation in this globally important ecosystem. Our findings may contribute to the understanding of soil nutrient status and nutrient interactions under changing climatic conditions.

## Materials and Methods

### Site description

**Figure 5:**
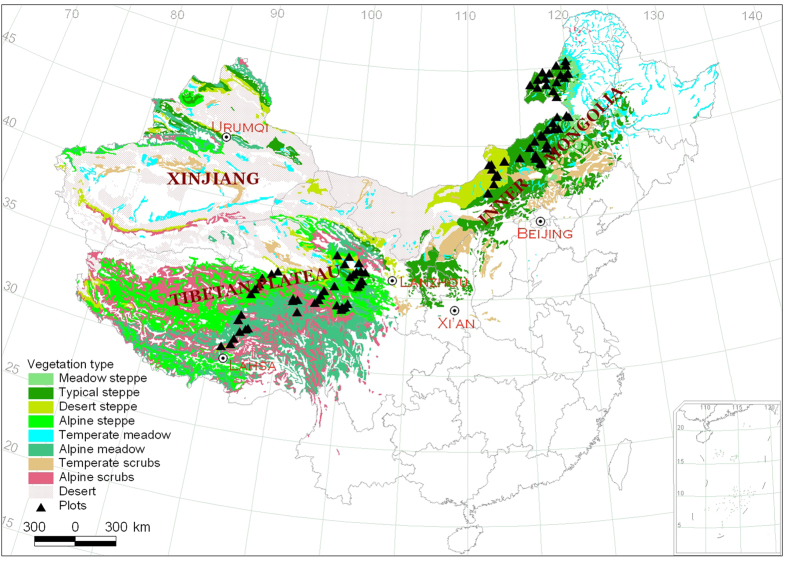
Sampling map of this study. Triangles represent sampling sites. The map was created using ArcGIS 10.2 (Esri, CA, http://www.esri.com).

This study was conducted in alpine grassland on the Tibetan Plateau and temperate grassland on the Inner Mongolian Plateau. A total of 80 sites were selected along temperature and precipitation gradients, extending latitudes from 30.31 to 50.19° N and longitudes from 90.80 to 120.12° E, along with altitudes from 553 to 5105 m (Table S2). Mean annual temperature (MAT) and mean annual precipitation (MAP) range from −5.8 to 4.1 °C and 148 to 604 mm, respectively (Table S2). Sites along the approximately 4,000 km transect represent natural grassland in these regions, including five main vegetation types: typical steppe, meadow steppe, desert steppe, alpine steppe and alpine meadow (Fig. 5).

### Soil and biomass survey

Soil sampling was split into two parts: schematic soil sampling by drilling at two depths (0–10 and 10–20 cm) for C, N and P analysis, as well as volumetric sampling using a standard container (100 cm^3^ in volume) at equal depths for soil bulk density and gravimetric water content determination. At each site five soil samples of each depth-increment were collected.

Soil samples were air-dried, all live root material was removed and the remaining soil was grounded using a ball mill (NM200, Retsch, Germany). Analyses for soil total nitrogen and soil bulk density have been described in detail previously^55^. Soil available nitrogen was determined photometrically from on-site KCl extractions using a continuous flow analyser (SAN Plus, Skalar, Netherlands)^20^. Soil total phosphorus was measured from a H_2_SO_4_ and HClO_4_ acid digest using a phosphomolybdate blue method. For P extraction, soil samples were extracted with 0.5 m NaHCO_3_, filtered and analyzed for orthophosphate by reaction with acid molybdate and reduction with ascorbic acid^56^. Soil pH was determined in both 0.01 M CaCl_2_ and bi-distilled H_2_O potentiometrically, but only those values of water solution were used in the current study. Soil moisture was determined gravimetrically by taking the skeleton content into account. Area-based concentrations of soil N and P of the two depth increments were calculated by using the mass-based data with soil bulk density. In addition, at each site we harvested aboveground biomass in three plots (1 × 1 m^2^) and belowground biomass (0.5 × 0.5 m^2^) in three soil pits. Biomass was measured by oven-drying at 60 °C to a constant weight and weighting to the nearest 0.1 g.

Climate data for each site was calculated based on linear models using latitude, longitude and altitude as predictors from 55-yr average temperature and precipitation records (1951–2005) at 680 climate stations across China^57^. The calculation of potential evapotranspiration (PE) and actual evapotranspiration (AE) were based on Thornthwaite’s method^58^.

### Statistical analysis

By using mean and standard errors, all soil variables at each site were averaged to obtain site-level estimates for statistical analysis. Comparisons of soil N and P concentrations as well as their ratios were made among different biogeographic regions, vegetation types and soil layers. The region and soil depth effects were tested using t-tests while one-way ANOVA with a Tukey’s *post hoc* test was used to test the vegetation type effect. The relationships of independent variables (MAT and MAP) with the dependent variables (soil N and P and their stoichiometric ratios) were explored by using linear regressions. For pairwise relationships between total N and P and between available N and P, SMA slopes and correlation coefficients of two site assemblages (at grassland type and regional scales) were plotted against MAT. The slopes and correlation coefficients of each assemblage were calculated using the ‘smatr’ package for R. The total variance of soil total and available N and P was partitioned into climate (MAT, MAP, PE, AE), soil (pH and moisture) and plant community (above- and below-ground biomass) components. All terms were estimated by using the hierarchical partitioning method in ‘hier.part’ package available within R, in order to identify the most likely causal factors for variation in soil N and P. To address how the explanatory variables screened out by hierarchical partitioning affected soil N and P availability both directly and indirectly (i.e. act through other variables), structural equation modelling (SEM) was used to account for the role of each variable. Overall fit of SEM was evaluated using model Chi-square (values closer to zero indicate a better fit), *P*-values (if *P* > 0.05, then no paths are missing and the model is a good fit), comparative fit index (CFI, a value of over 0.9 generally indicating acceptable model fit) and root mean square error of approximation (RMSEA, conventionally a good model is accurate when RMSEA < 0.05). The SEM model was fitted using ‘sem’ package in R. All statistical analyses were performed using software R 3.2.2.

## Additional Information

**How to cite this article:** Geng, Y. *et al*. Increasing temperature reduces the coupling between available nitrogen and phosphorus in soils of Chinese grasslands. *Sci. Rep.* **7**, 43524; doi: 10.1038/srep43524 (2017).

**Publisher's note:** Springer Nature remains neutral with regard to jurisdictional claims in published maps and institutional affiliations.

## Supplementary Material

Supplementary Information

## Figures and Tables

**Table 1:** Soil total and available N and P concentrations and their stoichiometric ratios in Inner Mongolian and the Tibetan grasslands.

	Inner Mongolia	Tibet	Overall
Total N (mg g^−1^)	1.72 ± 0.15 (39) a	4.40 ± 0.57 (43) b	3.13 ± 0.34
Available N (mg kg^−1^)	6.39 ± 1.19 (39) a	6.48 ± 0.87 (41) a	6.42 ± 0.71
Total P (mg g^−1^)	0.34 ± 0.02 (31) a	0.57 ± 0.02 (46) b	0.47 ± 0.02
Available P (mg kg^−1^)	2.91 ± 0.19 (33) a	8.23 ± 0.72 (46) b	5.91 ± 0.57
Total N:P ratio	5.2 ± 0.3 (32) a	6.9 ± 0.7 (42) b	6.3 ± 0.5
Available N:P ratio	2.3 ± 0.2 (32) b	0.8 ± 0.1 (40) a	1.5 ± 0.2

Values are means ± 1 SE; different letters between two items in a row indicate statistical significance at *P* < 0.05. Number of observations are show in parentheses.

**Table 2:** Percentages of variations in soil N and P concentrations explained by climatic and soil variables and plant biomass.

	Total N		Available N		Total P		Available P	
	0–10 cm	10–20 cm	0–10 cm	10–20 cm	0–10 cm	10–20 cm	0–10 cm	10–20 cm
MAT	16.63	11.67	11.11	10.56	10.01	8.85	10.79	9.11
MAP	13.72	7.19	10.21	9.43	13.11	13.51	20.56	24.43
PE	4.38	9.36	6.53	4.62	5.15	5.28	14.48	12.92
AE	10.72	9.95	18.05	19.69	18.93	10.35	16.45	5.13
pH	2.37	5.11	4.17	2.83	13.87	23.97	9.25	20.29
Soil moisture	10.16	26.29	6.07	19.64	9.54	14.54	3.46	17.75
Aboveground biomass	18.4	11.86	17.44	8.78	10.46	9.17	8.93	3.71
Belowground biomass	23.62	18.56	26.42	24.45	18.92	14.32	16.07	6.66

MAT, mean annual temperature; MAP, mean annual precipitation; PE, potential evapotranspiration; AE, actual evapotranspiration.
