# Supplementary material for: Increasing temperature reduces the coupling between available nitrogen and phosphorus in soils of Chinese grasslands
Source: Sci Rep. 2017 Mar 7;7:43524. doi: 10.1038/srep43524 (PMC5339893; doi:10.1038/srep43524)

Supplementary information

Increasing temperature reduces the coupling between available nitrogen and phosphorus in soils of Chinese grasslands

Yan Geng[1*], Frank Baumann[3], Chao Song[4], Mi Zhang[2], Yue Shi[1], Peter Kühn[3], Thomas Scholten[3], Jin-Sheng He[1, 2**]

[1]*Department of Ecology, College of Urban and Environmental Sciences, and Key Laboratory for Earth Surface Processes of the Ministry of Education, Peking University, 5 Yiheyuan Rd., Beijing 100871, China*

[2] *Key Laboratory of Adaptation and Evolution of Plateau Biota, Northwest Institute of Plateau Biology, Chinese Academy of Sciences, 23 Xinning Rd., Xining 810008, China*

[3]*Department of Geoscience, Soil Science and Geomorphology, University of Tuebingen, Ruemelinstrasse 19-23, 72070 Tuebingen, Germany*

[4]*Odum School of Ecology, University of Georgia, 140 E Green St, Athens, GA 30602, USA*

Contents of this file:

Table S1-S2

Figure S1-S2

Table S1. Mass and area- based concentrations of soil N and P at depths of 0-10 cm and 10-20 cm in Inner Mongolia and the Tibetan Plateau. Values are means ±1 SE; different letters between two items in a row indicate statistical significance between the two regions at $P < 0.05$. For available N and P, concentration units based on mass and area are mg kg^{-1} and g m^{-2}, respectively.

		Inner Mongolia	Tibet	Overall	Inner Mongolia	Tibet	Overall
		Mass based (mg g^{-1})			Area based (kg m^{-2})		
Total N	0-10cm	1.95±0.18 a	4.81±0.55 b	3.45±0.34	0.24±0.02 a	0.35±0.03 b	0.29±0.02
	10-20cm	1.36±0.11 a	3.89±0.53 b	2.73±0.32	0.17±0.01 a	0.27±0.02 b	0.22±0.02
Available N	0-10cm	8.13±1.79 a	7.59±0.80 a	7.85±0.95	0.59±0.19 a	0.58±0.05 a	0.78±0.10
	10-20cm	4.89±0.82 a	5.06±0.92 a	4.98±0.61	0.33±0.10 a	0.36±0.03 a	0.49±0.05
Total P	0-10cm	0.33±0.02 a	0.60±0.02 b	0.49±0.02	0.040±0.002 a	0.052±0.003 b	0.050±0.002
	10-20cm	0.30±0.02 a	0.54±0.02 b	0.44±0.02	0.038±0.002 a	0.048±0.003 b	0.044±0.002
Available P	0-10cm	3.34±0.23 a	9.88±0.88 b	7.14±0.63	0.42±0.03 a	0.76±0.06 b	0.61±0.04
	10-20cm	2.23±0.13 a	6.23±0.55 b	4.56±0.39	0.29±0.02 a	0.50±0.04 b	0.41±0.03

Table S2. Description of the study regions. Means of altitude, mean annual temperature (MAT), mean annual precipitation (MAP), potential evapotranspiration (PE), actual evapotranspiration (AE), averaged topsoil (0-20 cm) bulk density, pH and moisture of the sampling sites are shown *in parentheses.*

	Inner Mongolia	Tibet
No. of sites	36	44
Longitude (°E)	111.83-120.12	90.80-101.48
Latitude (°N)	41.79-50.19	30.31-37.28
Altitude (m)	553-1421 (899)	2925-5105 (4094)
MAT (^{o}C)	-2.6-4.1 (0.6)	-5.8-2.6 (-2.2)
MAP (mm)	148-436 (298)	218-604 (442.5)
PE (mm)	429-608 (528)	299-470 (360)
AE (mm)	148-435 (301)	171-423 (332)
Bulk density (g cm^{-3})	0.94-1.63 (1.30)	0.25-1.47 (0.95)
pH	5.73-8.17 (6.89)	5.18-7.56 (6.80)
Soil moisture (%)	2.04-16.10 (7.29)	0.73-68.10 (21.00)

Figure S1. Soil N and P in relation to mean annual precipitation. The solid lines represent significant linear regressions (*P* < 0.05) and the short dashed lines represent insignificant regressions (*P* > 0.05). The values of soil N and P are log-transformed. MAP, mean annual precipitation.

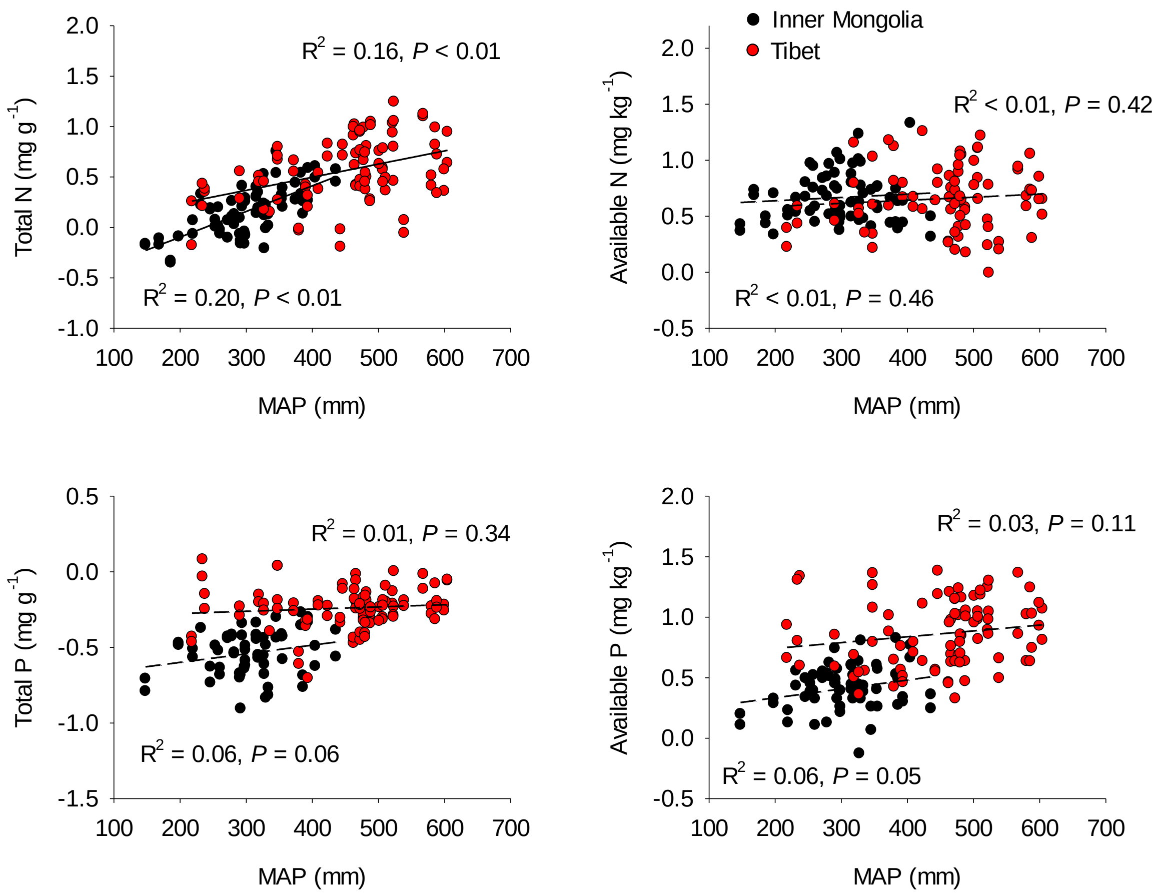

Figure S2. Ratios of soil total N:P, available N:P, available: total N and available: total P in relation to mean annual precipitation. The short dashed lines represent fitted regressions. MAP, mean annual precipitation.

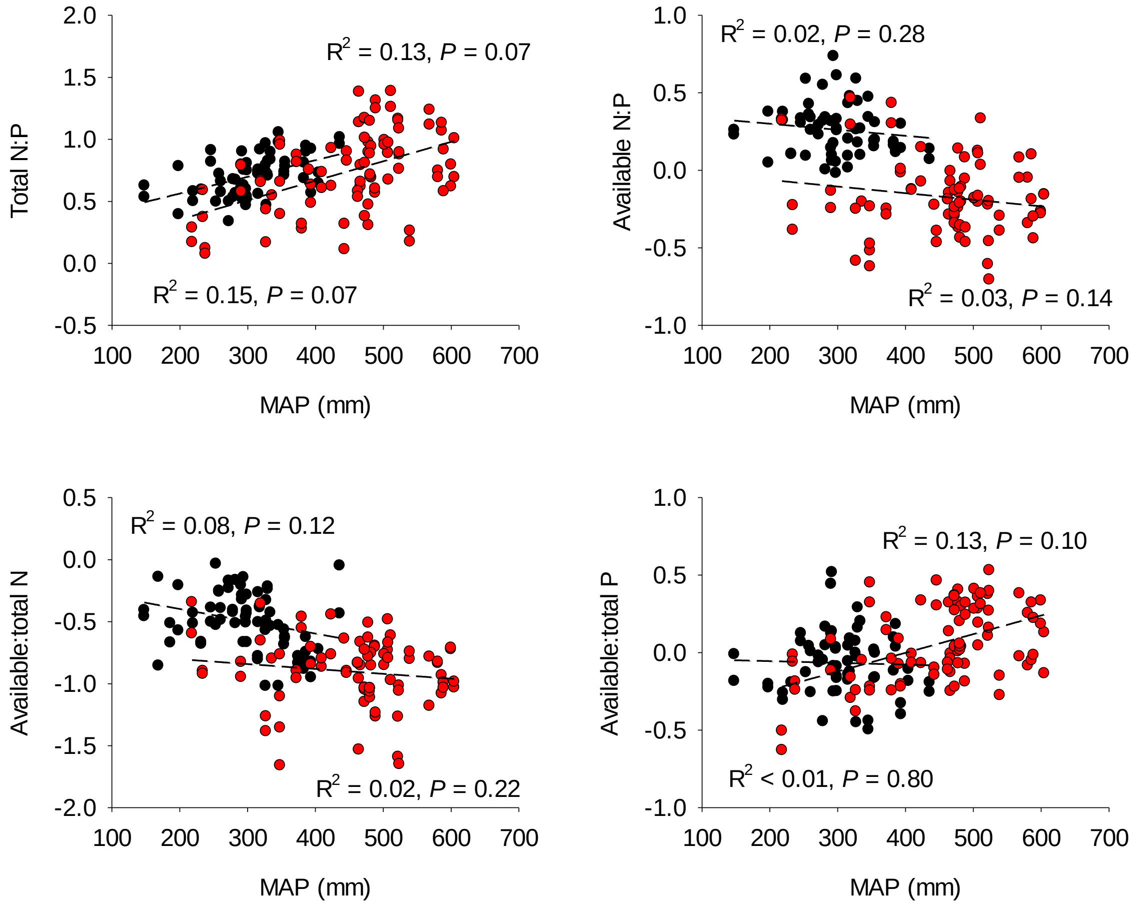

Supplement: Supplementary Information [file srep43524-s1.pdf]
